# oxLDL-Induced Trained Immunity Is Dependent on Mitochondrial Metabolic Reprogramming

**DOI:** 10.20900/immunometab20210025

**Published:** 2021-06-30

**Authors:** Laszlo A. Groh, Anaisa V. Ferreira, Leonie Helder, Charlotte D. C. C. van der Heijden, Boris Novakovic, Els van de Westerlo, Vasiliki Matzaraki, Simone J. C. F. M. Moorlag, L. Charlotte de Bree, Valerie A. C. M. Koeken, Vera P. Mourits, Samuel T. Keating, Jelmer H. van Puffelen, Alexander Hoischen, Leo A. B. Joosten, Mihai G. Netea, Werner J. H. Koopman, Niels P. Riksen

**Affiliations:** 1 Department of Internal Medicine, Radboud University Medical Center, Nijmegen 6525 GA, The Netherlands; 2 Instituto de Ciências Biomédicas Abel Salazar (ICBAS), Universidade do Porto, Porto 4050-313, Portugal; 3 Department of Anesthesiology, Radboud University Medical Center, Nijmegen 6525 GA, The Netherlands; 4 Complex Disease Epigenetics, Murdoch Children’s Research Institute and Department of Paediatrics, University of Melbourne, Parkville, VIC 3052, Australia; 5 Department of Biochemistry, Radboud Institute for Molecular Life Sciences, Radboud Center for Mitochondrial Medicine, Radboud University Medical Center, Nijmegen 6500 HB, The Netherlands; 6 Department of Computational Biology for Individualised Infection Medicine, Centre for Individualised Infection Medicine (CiiM) & TWINCORE, joint ventures between the Helmholtz-Centre for Infection Research (HZI) and the Hannover Medical School (MHH), Hannover 30625, Germany; 7 Department of Biology, University of Copenhagen, Copenhagen DK-2200 , Denmark; 8 Department for Health Evidence, Radboud University Medical Center, Nijmegen 6525 EZ, The Netherlands; 9 Department of Human Genetics, Radboud University Medical Center, Nijmegen 6525 GA, The Netherlands; 10 Department of Medical Genetics, Iuliu Haţieganu University of Medicine and Pharmacy, Cluj-Napoca 400349, Romania; 11 Department for Genomics & Immunoregulation, Life and Medical Sciences Institute (LIMES), University of Bonn, Bonn 53127, Germany

**Keywords:** monocyte, trained immunity, oxidized low-density lipoprotein, mitochondria, metabolic reprogramming

## Abstract

Following brief exposure to endogenous atherogenic particles, such as oxidized low-density lipoprotein (oxLDL), monocytes/macrophages can adopt a long-term pro-inflammatory phenotype, which is called trained immunity. This mechanism might contribute to the chronic low-grade inflammation that characterizes atherosclerosis. In this study, we aim to elucidate immunometabolic pathways that drive oxLDL-induced trained immunity. Primary isolated human monocytes were exposed to oxLDL for 24 h, and after five days stimulated with LPS to measure the cytokine production capacity. RNA-sequencing revealed broad increases in genes enriched in mitochondrial pathways after 24 h of oxLDL exposure. Further omics profiling of oxLDL-trained macrophages via intracellular metabolomics showed an enrichment for tricarboxylic acid (TCA) cycle metabolites. Single cell analysis revealed that oxLDL-trained macrophages contain larger mitochondria, potentially likely linked to increased oxidative phosphorylation (OXPHOS) activity. Co-incubation with pharmacological blockers of OXPHOS inhibited oxLDL-induced trained immunity. The relevance of OXPHOS was confirmed in a cohort of 243 healthy subjects showing that genetic variation in genes coding for enzymes relevant to OXPHOS correlated with the capacity of monocytes to be trained with oxLDL. Interestingly, OXPHOS appears to play an important role in the increased cytokine hyperresponsiveness by oxLDL-trained macrophages. The TCA-cycle can also be fuelled by glutamine and free fatty acids, and pharmacological blockade of these pathways could prevent oxLDL-induced trained immunity. This study demonstrates that the mitochondria of oxLDL-trained macrophages undergo changes to their function and form with OXPHOS being an important mechanism for trained immunity, which could unveil novel pharmacological targets to prevent atherogenesis.

## Introduction

Cardiovascular diseases (CVD) are amongst the leading causes of mortality worldwide. The primary pathophysiological process giving rise to CVD is atherosclerosis, which is characterized by the build-up of lipid-laden plaques within arteries. The most abundant immune cell type within plaques are macrophages, primarily originating from circulating monocytes [1]. These macrophages secrete cytokines and chemokines that contribute to a persistent state of inflammation in the arterial wall, contributing to the progressive development of atherosclerosis. Targeting of general inflammatory processes in patients with atherosclerotic CVD, with either the non-specific anti-inflammatory drug colchicine or the interleukin-1β (IL-1β) antibody canakinumab was recently proven to be effective for the prevention of future CVD [2,3].

We recently described a novel immunological mechanism that might contribute to the persistent arterial wall inflammation that drives atherosclerotic CVD termed trained immunity. This is defined as the ability of innate immune cells, such as monocytes and macrophages, to retain a de facto immunological memory of previous immune challenges, which translates into a long-lasting hyperresponsive phenotype characterized by an augmented cytokine response following subsequent encounters with similar or unrelated stimuli [4]. In the context of infectious diseases, this mechanism can offer improved protection of the host against recurrent infections, which most likely explains the protective effect of Bacillus Calmette-Guérin (BCG) vaccination against unrelated infections [5].

In the context of atherosclerosis, however, trained immunity is thought to be maladaptive by giving rise to the chronic low-grade atherosclerotic inflammation [4]. Importantly, trained immunity is not only induced by micro-organisms, but also by endogenous atherogenic stimuli, such as oxidized low-density lipoprotein (oxLDL), lipoprotein (a), and catecholamines [6,7]. Additional indications that trained immunity might contribute to atherosclerosis include the observations that monocytes isolated from patients with risk factors for CVD, such as hypercholesterolemia, or from patients with established atherosclerosis, are characterized by an increased cytokine production capacity and accompanying markers of trained immunity [8,9]. As such, elucidation of the underlying cellular mechanisms of trained immunity could offer novel targets for pharmacotherapy [10].

In most cells, glycolysis and mitochondrial oxidative phosphorylation (OXPHOS)-mediated ATP generation sustain cellular function and viability. The relative contribution of these ATP-generating pathways is flexible and becomes altered during metabolic impairment and changes in ATP demand [11,12]. In addition, both pathways can be simultaneously upregulated and/or activated to meet increased overall cellular energy demands. Trained immunity is accompanied by a broad reprogramming of the macrophage intracellular metabolic pathways as well as by epigenetic modifications, dual processes which are closely intertwined [13,14]. With respect to the metabolic reprogramming, most data are derived from training with β-glucan, a cell wall component of *Candida albicans*, and the tuberculosis vaccine BCG. For both stimuli, trained immunity critically depends on activation of glycolysis [15,16]. In addition, these training stimuli increase the oxygen consumption rate (OCR) compatible with parallel OXPHOS upregulation [15,17]. Interestingly, for β-glucan induced trained immunity this effect appeared to be dose-dependent [16]. In addition, it has been reported that β-glucan-induced trained immunity depends on glutamine fuelling of the tricarboxylic acid (TCA) cycle, paralleled by accumulation of fumarate [18], and activation of the mevalonate synthase pathway [19]. In the case of oxLDL-induced trained immunity, it was likewise shown to be dependent on increased glycolysis [20], but detailed information on mitochondrial energy metabolism is still lacking. Therefore, in this study we aim to elucidate the role of mitochondrial metabolism in oxLDL-induced trained immunity.

## Materials and Methods

### Reagents

CCCP (C2759), Oligomycin (75351), BPTES (SML0601), and Etomoxir (E1905) were all obtained from Sigma-Aldrich (St. Louis, MO, USA). Nicotinamide (481907) was obtained from Calbiochem (San Diego, CA, USA). SRT1720 (S1129) was obtained from Selleckchem (Munich, Germany). Doxycycline (Vibramycin^®^ SF) was obtained from Pfizer (New York City, NY, USA). Escherichia coli lipopolysaccharide (LPS; serotype O55:B5; Sigma-Aldrich (St. Louis, MO, USA)) was further purified as described previously [21]. OxLDL was prepared from human isolated LDL as previously described [22].

### PBMC and Monocyte Isolation

Peripheral blood mononuclear cells (PBMCs) were isolated from blood of healthy donors after they provided written informed consent (Sanquin Blood Bank, Nijmegen, The Netherlands) by dilution in PBS and density-gradient centrifugation using Ficoll-Paque (GE healthcare, Chicago, IL, USA). PBMCs were washed 3 times with cold PBS and resuspended in RPMI 1640 Medium (“Dutch modification” containing 11 mM glucose; Thermo-Fischer, Waltham, MA, USA) supplemented with 10 μg/mL gentamicin, 2 mM GlutaMAX and 1 mM pyruvate. Monocytes were isolated from PBMCs as described previously [23] using hyper-osmotic Percoll (Sigma-Aldrich, St. Louis, MO, USA). Cells were resuspended in RPMI 1640 medium and counted using a Beckman Z1 Coulter Particle counter.

### In Vitro Training of Adherent Monocytes

Training of adherent monocytes was performed as described previously [24]. In brief, after adhering for 1 h at 37 °C 5% CO_2_ and washing with warm PBS, monocytes were incubated with 200 μL RPMI 1640 medium only (negative control) or 10 μg/mL oxLDL for 24 h at 37 °C 5% CO_2_. Next, the stimuli were washed off with warm PBS, and changed to RPMI supplemented with 10% human serum, incubated at 37 °C 5% CO_2_ for 5 days with a medium refreshment on day 3. Cells were restimulated with 200 μL RPMI medium or 10 ng/mL LPS on day 6. After 24 h, supernatants were collected and stored at -20 °C until cytokines were measured.

### Cytokine Quantification

Levels of TNFα and IL-6 were measured in supernatants using the IL-6 and TNF-alpha DuoSet ELISA kits (R&D Systems, Minneapolis, MN, USA).

### Lactate Dehydrogenase (LDH) Measurements for Cell Viability

Analysis of LDH as a measure of cytotoxicity in cells incubated with oligomycin, CCCP, doxycycline, BPTES, Etomoxir, nicotinamide, and SRT1720 for 24 h was assessed at the specified concentrations in the supernatants by using a Cytotox 96 kit (Promega, Madison, WI, USA).

### RNA Isolation

Monocytes were isolated using magnetic activated cell sorting with MACS^®^ pan monocyte isolation (Miltenyi Biotec, 130-096-537, Cologne, Germany), and trained with oxLDL as described above. Cells were collected at the relevant timepoints, and harvested in RLT buffer (Qiagen, 79216, Hilden, Germany) before freezing at -80 °C. RNA was isolated using RNeasy mini columns (Qiagen, Hilden, Germany) according to the manufacturer’s instructions.

### RNA Sequencing and Differential Gene Expression Analysis

RNA sequencing library preparation was performed using the Quantseq 3’ mRNA-Seq Library Prep Kit-FWD from Lexogen (Lexogen, 015.96, Vienna, Austria) according to the manufacturer’s instructions. Sequencing of the libraries was performed on a NextSeq 500 instrument (Illumina, San Diego, CA, USA). Reads were mapped to a human reference genome (hg19) with Bowtie [25]. Quantification of gene expression levels as RPKM was performed using MMSEQ [26]. Differential gene expression analysis was performed with DESeq2 v1.22.0, with a fold change of >2.5 for genes with >5 RPKM considered differentially expressed. An average expression value was generated by combining the 3 donors for each condition per a timepoint. We then performed Gene Ontology analysis using the online platform Database for Annotation, Visualization, and Integrated Discovery (DAVID) in order to identify novel pathways of interest [27].

### Metabolomics Analysis

Approximately 1 × 10^7^ monocytes were trained with oxLDL (10 μg/mL) or unstimulated in 10 cm Petri dishes (Greiner, Alphen aan de Rijn, The Netherlands) in 10 mL RPMI 1640 volumes for 24 h, washed with warm PBS and incubated in normal culture medium at 37 °C, 5% CO_2_. Following 5 days in culture cells were washed 3 times with 75 mM ammonium carbonate in demi-water (pH 7.4; adjusted with formic acid), and snap frozen in liquid nitrogen. Intracellular metabolites were extracted with 70% ethanol heated to 70°C, supernatants were collected and stored at -80 °C [28]. Untargeted analysis of metabolites was performed by flow injection-time-of-flight mass spectrometry on an Agilent 6550 QTOF Instrument operated in negative mode 4 GHz, high resolution in a mass to charge (*m*/*z*) range of 50–1000, as described in [28]. Data was analyzed using the online platform MetaboAnalyst version 4.0, Metabolite Set Enrichment analysis (MSEA) was performed on a list of mass spectrometry peaks and fold changes derived from the comparison of oxLDL trained to naïve macrophage metabolites. This algorithm infers metabolic functionality from a list of mass spectrometry peaks with the use of *a priori* knowledge of metabolic pathway organization, allowing for the detection of subtle shifts in metabolic processes without needing prior annotation [29,30]. Pathway analysis was performed using the manually curated MFN metabolite library original to the mummichog package.

### Fluorescence Microscopy Analysis Using TMRM

Monocytes were trained with 10 μg/mL of oxLDL for 24 h and then allowed to rest for an additional 5 days in RPMI 1640 supplemented with 10% human serum. Isolated macrophages were seeded at a density of 4 × 10^6^/dish on fibronectin-coated glass-bottomed culture dishes (FluoroDishes; #FD35-100; World Precision Instruments Ltd., Friedberg, Germany). Prior to microscopy analysis, macrophages were incubated (in the dark; humidified atmosphere; 95% air; 5% CO_2_, 25 min, 37 °C, in the absence/presence of oxLDL) with 15 nM tetramethylrhodamine methyl ester (TMRM; #T668; Life Technologies Thermo Fisher Scientific, Waltham, MA, USA) diluted from a DMSO-dissolved stock solution. Directly following this incubation, the cells were placed on the stage of a fully motorized inverted microscope (Carl Zeiss, Jena, Germany; described in: [31]). Fluorescence images were acquired using an 63× objective (Carl Zeiss, Breda, The Netherlands), 540 nm excitation light delivered by a monochromator (TILL Photonics, Gräfelfing, Germany), a 560 nm dichroic mirror (#XF2017; Omega, Brattleboro, VT, USA), a 656 nm long pass emission filter (XF3085; Omega) and a CoolSNAP HD camera (Roper Scientific, Evry Cedex, France). Images were acquired within 15 min after the end of the TMRM staining and in the continuous presence of 15 nM extracellular TMRM. Mitochondrial TMRM intensity and morphology parameters were quantified using an automated method described previously [32]. Only objects with a size >10 pixels were included in the analysis.

### MitoSOX and TMRE Staining and Flow Cytometry Analysis

Mitochondrial reactive oxygen species (ROS) levels and mitochondrial membrane potential (ΔΨ), were determined after stimulation with oxLDL (at 2 h, 24 h and 6 days) by flow cytometry analysis of MitoSOX red (Invitrogen, #M36008, Waltham, MA, USA) and TMRE (Sigma-Aldrich, 87917, St. Louis, MO, USA), respectively. Adherent monocytes were detached with cold PBS and approximately 500,000 cells were stained by adding 250 μL of 5 μM MitoSOX red or 1 nM TMRE in HBSS + 1 mM CaCl_2_ or RPMI 1640, respectively. Next, cells were incubated at 37 °C for 30 min in the dark and immediately analysed by flow cytometry (Cytoflex, Beckman Coulter, Indianapolis, IN, USA). Data acquisition and analysis were performed using the Kaluza 2.1 software. Cell aggregates were excluded based on the forward scatter (FSC)-height versus FSC-area plot and the monocyte/macrophage population was gated according to FSC and side scatter (SSC) to remove doublets and cellular debris.

### Genetic Analysis

#### Genotyping, quality control and imputation of the 300BCG cohort

To investigate if genetic variation in genes encoding for mitochondrial enzymes impact on oxLDL-induced trained immunity, we performed genotyping in a cohort of 243 healthy individuals that participated in the 300BCG cohort (NL58553.091.16), where we isolated PBMCs and performed the trained immunity experiment with oxLDL as described above [33]. DNA samples of individuals were genotyped using the commercially available SNP chip, Infinium Global Screening Array MD v1.0 from Illumina. Opticall 0.7.0 with default settings was used for genotype calling [34]. Samples with a call rate ≤ 0.99 were excluded, as were variants with a Hardy-Weinberg equilibrium (HWE) ≤ 0.0001, and minor allele frequency (MAF) ≤ 0.1. Strands of variants were aligned and identified against the 1000 Genome reference panel using Genotype Harmonizer [35]. One sample was excluded from the pre-imputed dataset due to high relatedness. We then imputed the samples on the Michigan imputation server using the human reference consortium (HRC r1.1 2016) as a reference panel, and we filtered out genetic variants with an *R*_2_ < 0.3 for imputation quality [36]. We identified and excluded 17 genetic outliers. We selected 4,296,841 SNPs with MAF 5% for follow-up quantitative trait loci (QTL) mapping.

#### Training QTL mapping in 300BCG

Both genotype and in vitro cytokine data on innate memory responses induced by oxLDL was obtained for a total of 243 individuals. Three samples were excluded due to medication use (of which one was identified as a genetic outlier), and one sample due to onset of type 1 diabetes during the study. Few cytokine measurements under the detection limit (<39 pg/mL for TNF-α and <46.88 pg/mL for IL-6) were set to missing. First, the fold change of cytokine production between trained and non-trained cells was taken as a measurement for the magnitude of the trained immunity response. To check for normality of the fold change of cytokine production, we followed a visual inspection of the fold change of cytokine production using both raw and log-transformed data. Fold change of cytokine production followed non-Gaussian distribution before data transformation. Following quality check for cytokine distribution and after excluding genetic outliers, we mapped the log- transformed fold changes of cytokine production to genotype data using a linear regression model with age and sex as covariates to correct the distributions of fold change of cytokine production. R-package Matrix-eQTL was used for cytokine QTL mapping. Using a genome- wide significant threshold of *P* < 5 × 10^−8^, we did not observe genome-wide significant trained-immunity QTLs. We used a cut-off of *P* < 9.99 × 10^−3^ to identify suggestive QTL associations affecting trained immunity responses.

### Data Accessibility

RNA-sequencing data has been made available on the Gene Expression Omnibus, series number: GSE166238.

### Data Analysis

In vitro training for cytokine measurements on day 6 was performed with monocytes from 6 donors. Data are presented as mean + SEM. Statistical testing was performed by using the Wilcoxon matched-pairs signed rank test using GraphPad Prism 6. *P*-values < 0.05 were considered statistically significant.

## Results

### Mitochondrial Pathway-Linked Genes Are Upregulated by oxLDL Stimulation

To unveil metabolic pathways involved in oxLDL-induced trained immunity, we performed a comparative RNA-sequencing (RNA-seq) analysis of monocytes exposed to oxLDL or media only for 24 h followed by a 5-day wash-out period (trained macrophages). Differential gene expression in the oxLDL-trained cells was most pronounced at the 24 h timepoint (Supplementary Figure S1). Here, oxLDL-treated monocytes displayed 190 and 132 up- and down-regulated genes, respectively. Gene ontology (GO) analysis of the up-regulated genes revealed an enrichment in mitochondrial metabolic pathways (Table 1; Supplementary Table S1). In contrast, GO analysis of the down-regulated genes highlighted no mitochondria-linked pathways (Table 1). Additionally, KEGG pathway analysis was performed and similarly highlighted an increase in the expression of genes related to metabolism, with a particular emphasis on mitochondrial metabolism (Supplementary Table S2). These results demonstrate that 24 h of oxLDL stimulation induced upregulation of mitochondrial metabolic genes in monocytes. To better understand the functional impact of this upregulation, we performed metabolome analysis of oxLDL-trained macrophages. To this end, cell homogenates of trained macrophages were subjected to untargeted metabolomics and pathway analysis [29,30]. This highlighted the mitochondrial tricarboxylic acid (TCA) cycle as the most upregulated pathway, as well as increases in other mitochondria-linked pathways such as alanine/aspartate metabolism, C5-branched dibasic acid metabolism and pyruvate metabolism (Table 2; Supplementary Figure S2). A role of TCA metabolism has previously been seen in other inducers of trained immunity [15,17,18,37]. Additionally, we have previously reported an increased OCR in oxLDL-trained macrophages [20], similarly suggesting a reliance on the TCA cycle and OXPHOS in oxLDL-trained macrophages.

### Mitochondria Are Enlarged in oxLDL Trained Macrophages

**Figure 1:**
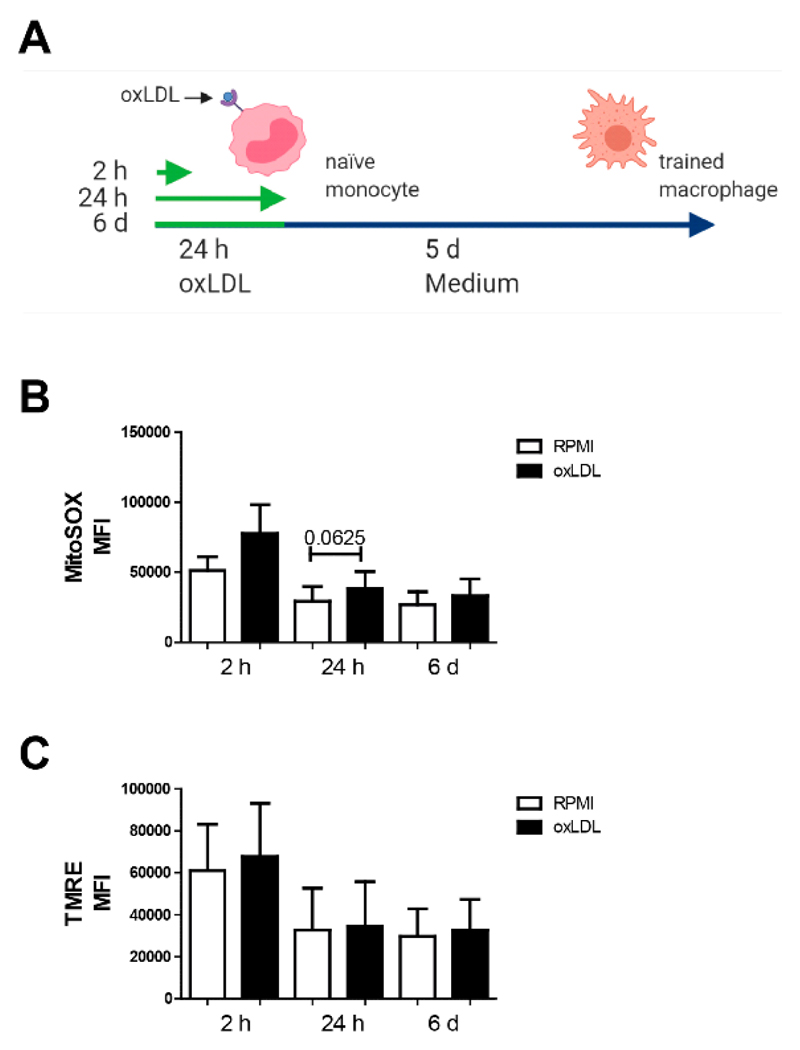
Early and sustained trend toward elevated mitochondrial ROS production in oxLDL trained macrophages. (**A**) Schematic overview of the sampling timepoints along the trained immunity protocol. (**B**) Analysis of mitochondrial physiology via flow cytometry using the mitochondrial dyes MitoSOX, and (**C**) TMRE. Data represented as mean fluoresce intensity (MFI) (mean + SEM, *n* = 6, Wilcoxon signed-rank test).

Alterations in mitochondrial functional state are often paralleled by changes in mitochondrial (ultra)structure, reactive oxygen species (ROS) level and redox homeostasis [38,39]. To determine if oxLDL affected these parameters, we studied its acute effect (2 h and 24 h incubation), as well as the persistent effect of a brief oxLDL exposure (24 h incubation followed by a 5 days washout period) (Figure 1A). For this purpose, cells stained with the ROS reporter molecule MitoSOX Red [40] or with the fluorescent cation TMRE (as a readout of mitochondrial membrane potential (ΔΨ)) were analysed using flow cytometry. Cellular MitoSOX oxidation (Figure 1B) and TMRE intensity (Figure 1C) were not significantly affected by oxLDL exposure at any time point. Of note, MitoSOX oxidation showed a mild, non-significant, increase after 2 h and 24 h of oxLDL exposure.

**Figure 2:**
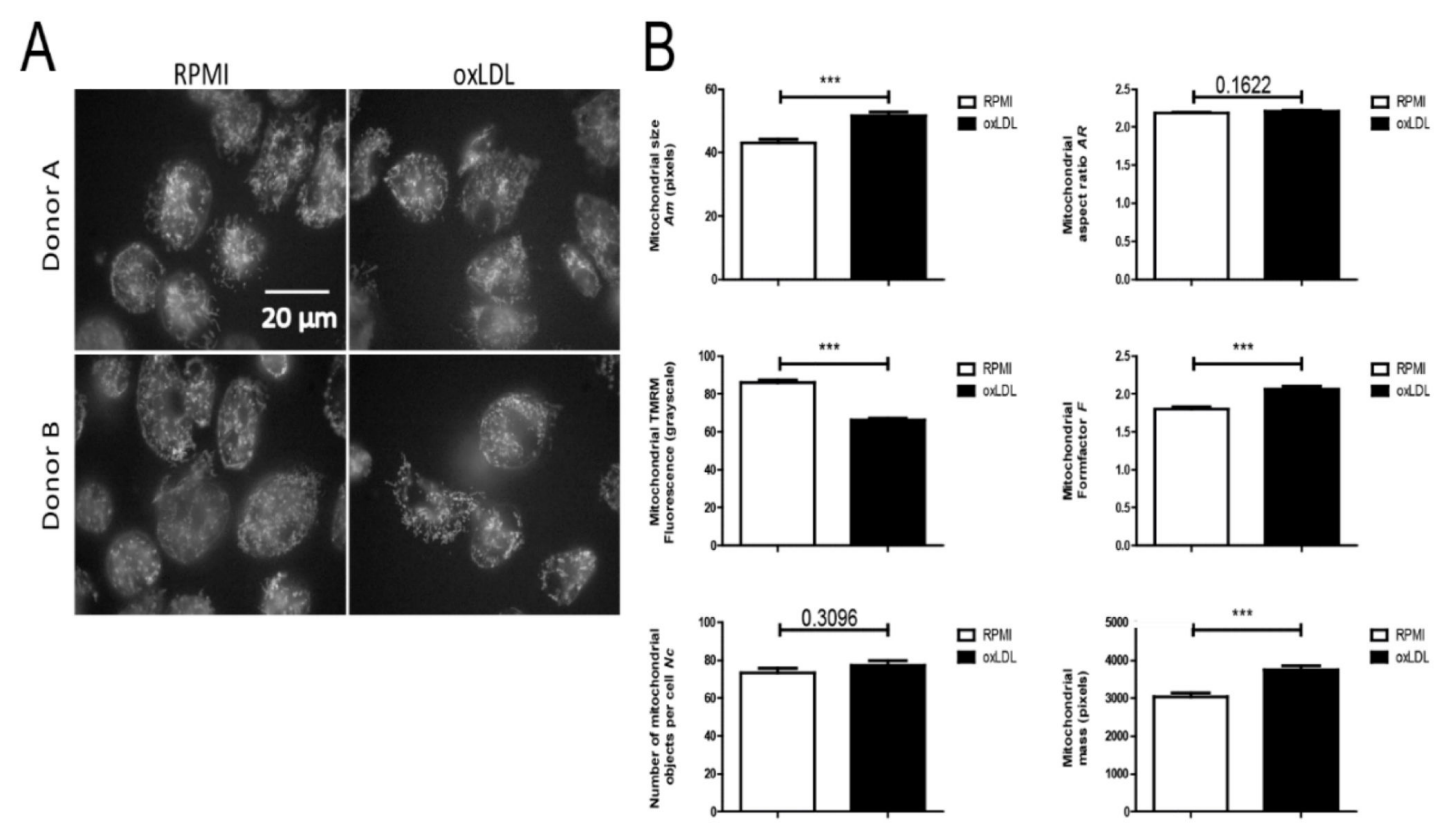
oxLDL trained macrophages display an increased mitochondrial mass and mitochondrial membrane potential depolarisation. Monocytes were incubated with medium (RPMI) or oxLDL for 24 h followed by washing and recovery for 5 days. Mitochondria were stained using the fluorescent cation TMRM and visualized by fluorescence microscopy. (**A**) Typical examples of TMRM-stained cells. (**B**) Image quantification was applied to determine mitochondrial size (*A*_m_), mitochondrial formfactor (*F*; a combined measure of mitochondrial length and degree of branching), mitochondrial aspect ratio (*A*_R_; a measure of mitochondrial length), the number of mitochondrial objects per cell (*N*_c_), mitochondrial mass (being the product of *A*_m_ and *N*_c_) and mitochondrial TMRM fluorescence intensity (a measure of ΔΨ) (From 2 donors; number of cells for RPMI *n* = 142, and oxLDL *n* = 149, *p < 0.05, **p < 0.01, ***p < 0.001, Mann-Whitney *U* test).

To obtain subcellular information, we used TMRM staining and live-cell fluorescence microscopy [32,41] to quantify mitochondrial morphology and ΔΨ in oxLDL-treated cells (2 donors; 24 h incubation followed by a 5 days washout period). To this end, fields of cells (Figure 2A) were analysed to obtain quantitative readouts of: mitochondrial size (*A*_m_), formfactor (*F*; a combined measure of mitochondrial length and degree of branching), aspect ratio (*A*_R_; a measure of mitochondrial length), number of mitochondrial objects per cell (*N*_c_), mitochondrial mass (being the product of *A*_m_ and *N*_c_) and mitochondrial TMRM fluorescence intensity (a measure of ΔΨ). We observed that *A*_m_, *F* and mitochondrial mass were significantly increased, TMRM intensity was significantly decreased, and *A*_R_ and *N*_c_ were not affected in oxLDL-treated cells (Figure 2B). Collectively, these observations demonstrate that individual mitochondrial objects are larger and display a partially depolarized ΔΨ in oxLDL-treated cells. The latter suggest that the mitochondrial electron transport chain (ETC) complexes (CI-CIV) are less active and/or CV-mediated mitochondrial ATP production is more active in oxLDL-treated cells. Our previous findings showing increased OCR in oxLDL-trained macrophages [20], as well as greater enrichment of TCA cycle metabolites (Table 2; Supplementary Figure S2), suggest that the partial ΔΨ depolarization is due to increased CV activity.

### SNPs in Genes Relevant for OXPHOS and the TCA Cycle Correlate with oxLDL-Induced Trained Immunity in PBMCs

**Figure 3:**
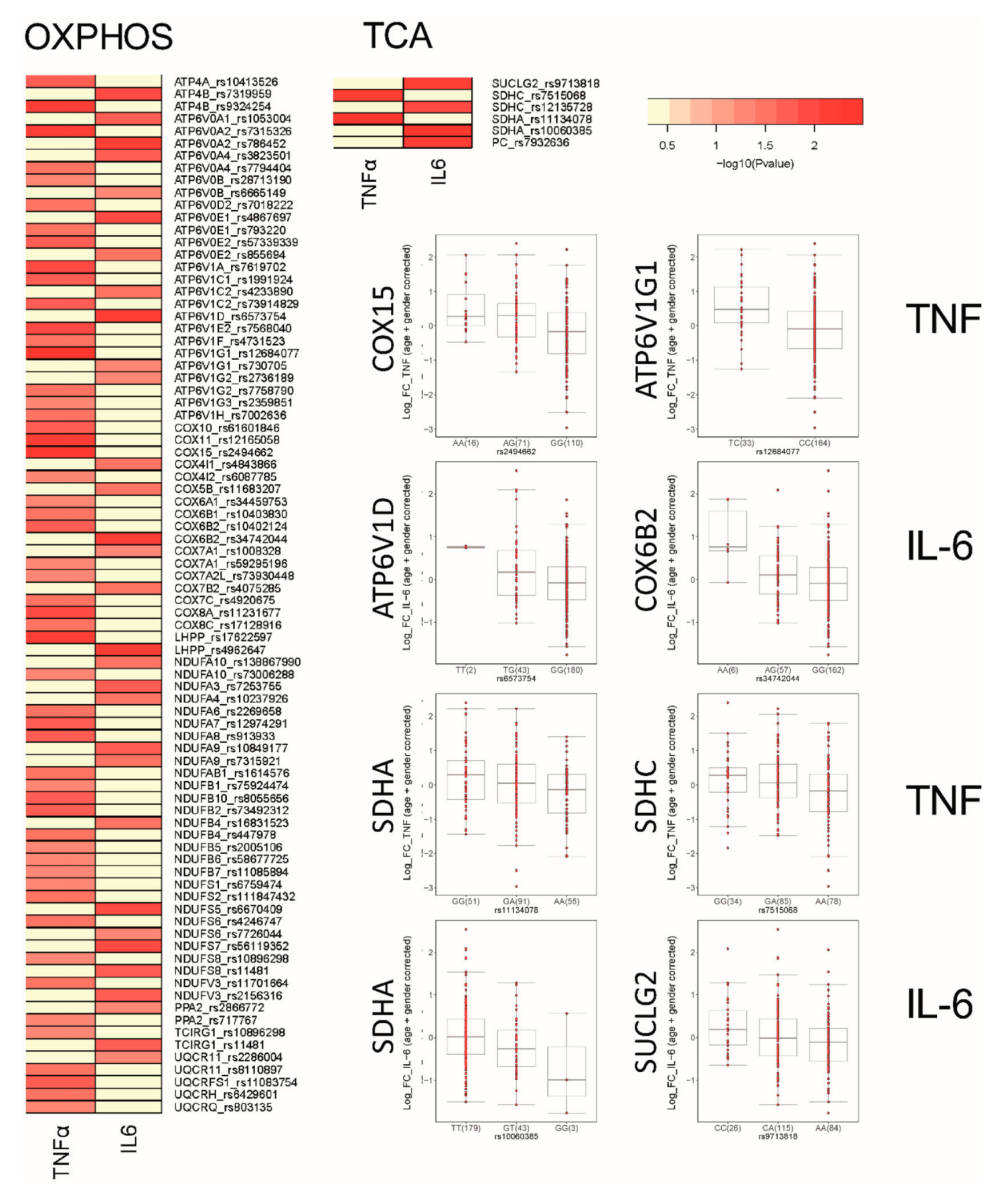
SNPs in TCA and OXPHOS genes are correlated with ex vivo trained of monocytes. Heatmap of the *P* values of association between SNPs mapped around genes involved in TCA and OXPHOS and the magnitude of cytokine production capacity by monocytes trained with oxLDL isolated from the 300BCG cohort. The colour legend for the heatmap indicates the range of *p* values from QTL mapping. Boxplots show the genotype-stratified cytokine levels for selected OXPHOS and TCA cycle loci.

Our data demonstrate an important role for mitochondria-mediated energy metabolism in oxLDL-trained macrophages. We further investigated this link by studying the contribution of genetic variation to individual inflammatory responses to oxLDL in monocytes. Using the 300BCG cohort, we investigated if single nucleotide polymorphisms (SNPs) mapped within a 250 kb window of genes encoding for enzymes involved in OXPHOS and the TCA cycle are correlated for TNFα and IL-6 production following ex vivo oxLDL training of PBMCs in 243 healthy volunteers. Although we did not observe genome-wide significant SNPs due to limited sample size, we identified numerous SNPs suggestively associated (*p* ≤ 9.99 × 10^−3^) with cytokine production following oxLDL-induced trained immunity (Figure 3A). We identified 84 SNPs in OXPHOS related genes and 7 SNPs in genes related to the TCA cycle. Among the strongest correlated SNPs were Cytochrome c oxidase assembly protein COX15 homolog (*COX15*; rs2494662, TNFα: *p* = 8.87 × 10^−5^) and Cytochrome c oxidase subunit VIb polypeptide 2 (*COX6B2*; rs34742044, IL-6: *p* = 3.12 × 10^−4^) which are genes relevant for Complex IV assembly and function respectively. Particularly strong SNPs were identified for various V-type proton ATPases which are ATPase H^+^ transporters, with particularly strong correlates being *ATP6V1D* (rs12684077, TNFα: *p* = 1.10 × 10^−4^) and *ATP6V1G1* (rs6573754, IL-6: *p* = 3.09 × 10^−4^). Additionally, we identified many SNPs in genes coding for subunits of NADH ubiquinone oxidoreductase, which comprise complex I of the electron transport chain.

Fewer TCA cycle genes were found to be correlated to cytokine production, however SNPs in the succinate dehydrogenase A gene were identified for both cytokines (*SDHA*; rs11134078, TNFα: *p* = 3.21 × 10^−3^ and rs10060385, IL-6: *p* = 4.76 × 10^−3^). SDHA is subunit of the second ETC complex (CII), which also is a key part of the TCA cycle that mediates the conversion of succinate to fumarate and FADH_2_. Collectively, these results underscore the importance of OXPHOS in driving the trained immunity response that follows brief exposure to oxLDL and supports our previous observation that there is strong interindividual variation in the magnitude of the trained immunity response to oxLDL [20].

### oxLDL-Induced Trained Immunity Is Effectively Suppressed by Pharmacological Blockade of Mitochondrial Function and Biosynthesis

**Figure 4:**
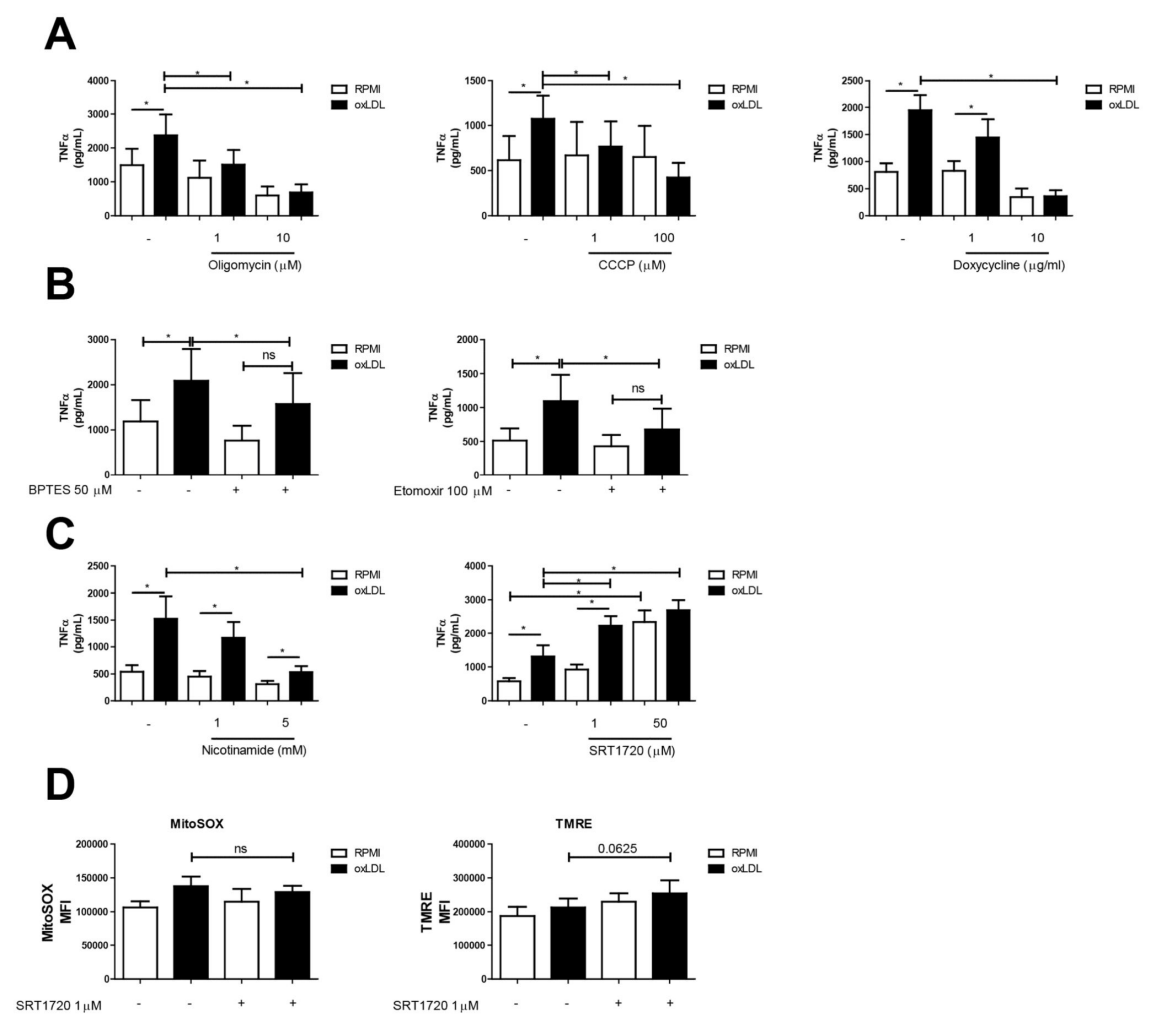
Pharmacological interference with mitochondrial function and development impacts macrophage training. Adherent monocytes were treated with oxLDL or medium supplemented with (**A**) Oligomycin (1 and 10 μM), CCCP (1 and 100 μM), Doxycycline (1 and 10 μg/mL) (**B**) BPTES (50 μM), Etomoxir (100 μM), (**C**) Nicotinamide (1 and 5 μM) or SRT1720 (1 and 50 μM) respectively. Cells were washed and rested in medium for 5 days. Cells were then restimulated with LPS (10 ng/mL) for 24 h and TNFα was measured in the supernatants (mean + SEM, *n* = 6, *p < 0.05, Wilcoxon signed-rank test). (**D**) Monocytes were trained with oxLDL + SRT1720. After 5 days resting in normal culture medium, cells were stained with MitoSOX or TMRE, and measured via flow cytometry (mean + SEM, *n* = 6 *p < 0.05, Wilcoxon signed-rank test).

Given the important role for the mitochondria in trained immunity, we investigated if modulating the energy production by mitochondria affects the ability of oxLDL-trained macrophages to mount an inflammatory response. To this end, we co-incubated monocytes with the CV inhibitor oligomycin and oxLDL during the first 24 h of the treatment protocol. This approach inhibited TNFα production in a dose-dependent manner (Figure 4A; left panel). Dissipation of the mitochondrial trans-inner membrane (MIM) proton gradient by a protonophore is another strategy to inhibit mitochondrial ATP generation. Similar to oligomycin, treatment with the protonophore CCCP also dose-dependently reduced TNFα production in oxLDL-trained macrophages (Figure 4A, centre panel). Inhibition of mitochondrial protein synthesis by the antibiotic doxycycline is a third strategy to hamper mitochondrial ATP generation [42]. Doxycycline lowered the level of TNFα production in oxLDL trained macrophages (Figure 4A, right panel) and prevented the induction of trained immunity altogether when monocytes were trained in the presence of 10 μg/mL of doxycycline.

We have demonstrated previously that glycolysis is important for giving rise to oxLDL induced trained immunity, while here we demonstrate that mitochondrial TCA metabolites are enriched in oxLDL trained macrophages. Since the TCA cycle is able to be fuelled by alternative nutrients, we were curious about potential other substrates that may be important for the metabolic adaption in oxLDL trained immunity. Given our metabolomics data (i.e., TCA upregulation), a candidate alternative substrate could be glutamine being converted into glutamate and entering the TCA cycle. Glutaminolysis is a metabolic pathway previously demonstrated to be involved in trained immunity induction by β-glucan [18]. In our metabolomics data, metabolites of this pathways showed a modest increase in enrichment (L-glutamine: log2(fc) = 0.088; L-glutamate: log2(fc) = 0.213; 2-oxoglutarate: log2(fc) = 0.180; 2-oxoglutaramate: log2(fc) = 0.087). We therefore sought to test whether inhibition of glutamine to glutamate conversion by glutaminase 1 would impact on oxLDL induced trained immunity. To this end we used the glutaminase 1 inhibitor BPTES. BPTES treatment lowered TNFα levels in LPS restimulated oxLDL-trained macrophages (Figure 4B; left panel). Another candidate substrate for OXPHOS is acyl-carnitine originating from fatty acids. Indeed vascular macrophages were shown previously to use fatty acids as an energy source [43]. The carnitine palmitoyltransferase-1 (CPT-1) inhibitor etomoxir effectively lowered TNFα levels in oxLDL-trained macrophages (Figure 4B; right panel).

We next sought to interfere with mitochondrial biogenesis by pharmacological manipulation of sirtuin-1 activity. Sirtuin-1 has been shown to initiate mitochondrial biogenesis [44], and therefore its inhibition provide evidence for whether the elevated OXPHOS and altered mitochondrial morphology within trained macrophages involves increased mitochondrial biogenesis. Sirtuin-1 inhibition via nicotinamide has been demonstrated to interfere with mitochondria homeostasis in macrophage cell lines [45]. Similar to the mitochondrial functional inhibitors (Figure 4A), nicotinamide lowered TNFα levels in oxLDL trained macrophages, while having only a minor effect on untrained macrophages (Figure 4C; left panel). To substantiate this finding, we performed parallel experiments with the resveratrol-derived sirtuin-1 activator SRT1720. Indeed 24 h treatment with SRT1720 augmented TNFα production in oxLDL-trained macrophages, and also in unstimulated cells where it resulted in a near four-fold increase in TNFα production following LPS restimulation (Figure 4C; right panel). Analysis of cellular MitoSOX oxidation revealed that SRT1720 treatment did not affect this parameter (Figure 4D; left panel). Flow cytometric analysis of TMRE fluorescence revealed a marginal increase in oxLDL trained macrophages treated with SRT1720, which reached borderline significance (Figure 4D; right panel). All inhibitors presented in this section showed no cellular cytotoxicity as measured by LDH release (data not shown).

## Discussion

The current study demonstrates that augmented OXPHOS, supported by an increase in mitochondrial size and mass, contributes to the cytokine hyperresponsiveness that characterizes the oxLDL-trained macrophage phenotype. These results add to the understanding of the pathophysiology of the chronic arterial wall inflammation in atherosclerosis, and provides novel potential pharmacological targets to dampen atherosclerotic CVD.

We have previously observed an increased OCR in oxLDL-trained macrophages [20], as well as in macrophages trained with BCG [15] and β-glucan [16]. Mitochondria in oxLDL-trained macrophages displayed a lower TMRM fluorescence signal, compatible with ΔΨ being less negative in these cells. The latter suggests that the mitochondrial electron transport chain (ETC) complexes (CI-CIV) are less active and/or CV-mediated mitochondrial ATP production is more active in oxLDL-treated cells. Given the previous observation of an increased OCR in oxLDL-trained macrophages, and the current observation of enriched TCA metabolites (Table 2; Supplementary Figure S2), we propose that the partial ΔΨ depolarization in oxLDL-treated cells is due to increased CV activity. The importance of OXPHOS in supporting trained immunity was made evident by the use of pharmacological inhibitors targeting mitochondrial function. Inhibition of OXPHOS with the use of oligomycin and CCCP curtailed the induction of trained immunity in response to oxLDL. These data further suggest that complex V hyperactivity is responsible for the partial depolarisation of mitochondria seen in oxLDL-trained macrophages. Doxycycline inhibits the function of mitochondrial ribosomes, interfering with effective mitochondrial function [46]. Doxycycline treatment was similarly shown to be a stringent inhibitor of trained immunity. Preferably, results from studies using pharmacological blockades are confirmed with genetic approaches. Unfortunately, this is notoriously difficult in primary human myeloid cells. Therefore, we used an alternative genetic approach, making use of interindividual genetic variation in metabolic enzymes in a large cohort of healthy subjects and the association to individual amplitude of trained immunity response to brief oxLDL-exposure. We showed that SNPs in genes relevant for OXPHOS and the TCA cycle correlated with the ability of oxLDL-trained PBMCs to produce TNFα and IL-6 following LPS restimulation. These SNPs occurred in genes that code for components of ETC complex I, II, and IV, further highlighting the importance of OXPHOS and the ETC in supporting the oxLDL-trained phenotype. In addition to confirming our pharmacological results, these results also provide a potential explanation for interindividual differences in the development of innate immune cell activation by atherogenic stimuli and atherosclerotic plaque development.

Supported by previous results [20], oxLDL-trained macrophages show an increased OCR suggesting that these cells rely on OXPHOS to meet their energy needs. Supporting this idea, metabolomics analysis here revealed that TCA cycle metabolites were enriched in oxLDL-trained macrophages. TCA cycle activity was previously demonstrated to play a role in β-glucan induced trained immunity, where glutaminolysis was increased and TCA cycle intermediates (including fumarate and itaconate) contributed to the pro-inflammatory phenotype in these macrophages [18,37]. In this system, fumarate was shown to modulate epigenetics by inhibition of the lysine demethylase KDM4 [18]. The current study revealed that the glutaminase-1 inhibitor BPTES inhibited oxLDL-induced trained immunity, supporting a mechanism in which glutamine-mediated OXPHOS fuelling is important in oxLDL-trained macrophages. Plaque macrophages have been shown to readily use fatty acids as an energy source [43]. Here we observed that the carnitine palmitoyltransferase-1 (CPT-1) inhibitor etomoxir inhibited oxLDL-induced trained immunity. CPT-1 is responsible for the final step in the conversion of fatty acids to acyl-carnitine prior to mitochondrial entry and TCA cycle fuelling. Therefore, the effect of etomoxir suggest that preventing acyl-carnitine formation inhibits oxLDL induced trained immunity, which is surprising since increased fatty acid oxidation (FAO) is classically a hallmark of anti-inflammatory macrophages [47]. Taken together, our results support a model in which (increased) OXPHOS activity is crucial for oxLDL-induced macrophage training and that this activity requires glutamine and/or FAs as a substrate.

Applying single cell analysis, we here demonstrate that oxLDL-trained macrophages contain larger mitochondria, which is probably linked to their increased OXPHOS activity [20]. In addition, gene ontology pathway analysis of upregulated genes in oxLDL-trained macrophages showed upregulation of pathways relevant to mitochondrial synthesis and function. It has previously been demonstrated that induction of mitochondrial fission in macrophages promotes atherosclerosis in mice [48], suggesting that alterations in mitochondrial fission might also be involved in the mitochondrial morphology changes in the current study. Moreover, stimulation of mitochondrial biogenesis could be (co)responsible for the observed increase in OXPHOS activity and mitochondrial size in oxLDL-trained macrophages. This biogenesis involves sirtuin-1 [44] and inhibition of the latter by nicotinamide serves to interfere with mitochondria homeostasis and alters macrophage function [45]. In the current study, nicotinamide treatment resulted in lower levels of measurable TNFα, while the Sirtuin activator SRT-1720 resulted in increased levels of TNFα and was also able to increase TNFα levels in naïve monocytes. This suggests that Sirtuin-1 activation might mediate the increase in OXPHOS activity and mitochondrial size in oxLDL-trained macrophages. Obviously, further studies are required to understand the exact role of Sirtuin-1 in our model system.

Oxidative stress in vascular lesions has been well-described in atherosclerotic diseases, and correlates well with disease severity. Most striking is the repeated finding of oxidized mitochondrial DNA in affected tissue [49,50]. Here we observed an apparent early increase in MitoSOX-oxidizing ROS levels in monocytes after 2 h of oxLDL stimulation. This increase reached near significance after 24 h of stimulation and remained apparently elevated 5 days after oxLDL was washed away (Figure 1B). Given the fact that MitoSOX is mitochondria-targeted, these results might suggest that increased mitochondrial size and OXPHOS activity is linked to increased mitochondrial generation of MitoSOX-oxidizing ROS. Compatible with the latter, evidence was provided that mitochondrial ROS levels are elevated in oxLDL-trained macrophages, and that lowering these levels during initial oxLDL stimulation sufficed to prevent trained immunity induction as measured by TNFα levels [51]. Whether this ROS is capable of inducing mtDNA oxidation, which is often observed in affected tissues during atherosclerosis [49], remains to be established.

A strength of this current study is the use of primary human monocytes and their integrated analysis using various techniques. On the other hand, this experimental model displays inherent heterogeneity leading sometimes to borderline-significant results that are not always directly translatable to the in vivo situation. However, by integrating the obtained results with data from the literature we were able to reach consistent conclusions. A point-of-interest is the fact that trained macrophages can display an increased cell size; this has mainly been described for β-glucan, but also oxLDL-trained macrophages showed a slight increase in cell size [24]. In contrast, oxLDL-training in the experimental design used in our current study does not increase cell numbers [24]. Other findings however, strongly argue against an increased cell size affecting our results: microscopy showed that oxLDL-trained cells had larger mitochondria but that the number of mitochondria per well was unchanged. In addition, our RNA-seq and genetic cohort analysis are not influenced by cell size while still offering the conclusion that mitochondrial function per se is important for oxLDL trained immunity.

## Conclusions

The current study provides evidence that oxLDL-trained macrophages display increased OXPHOS activity and contain larger mitochondria. Importantly, we here demonstrate that functional mitochondria, and in particular OXPHOS activity, are involved in the inflammatory response during oxLDL-induced trained immunity in macrophages. We further observed that glutamine and fatty acids, which can fuel the TCA-cycle, may further be important for trained immunity induction by oxLDL. Given the fact that MitoSOX is mitochondria-targeted, our results might suggest that increased mitochondrial size and OXPHOS activity are linked to increased mitochondrial ROS production. Finally, our results suggest that functional mitochondria are critical for cytokine hyperresponsiveness, which characterizes the trained macrophage phenotype.

## Supplementary Material

The supplementary materials are available online at https://doi.org/10.20900/immunometab20210025.

supplementary materials

## Figures and Tables

**Table 1:** Gene ontology analysis shows an increase in genes related to mitochondria physiology and function 24 h after oxLDL treatment. Tables showing the pathways of the most up and down regulated genes following 24 h of oxLDL (10 μg/mL) treatment (differential gene expression calculated against unstimulated controls; *n* = 3).

**RNA_seq Up 24 h oxLDL**			
Term Name	Raw *P*.Value	FDR *Q*-Value	Count
extracellular exosome	2.37 × 10^−18^	5.22 × 10^−16^	77
Mitochondrion	2.15× 10^−8^	2.36 × 10^−6^	37
mitochondrial inner membrane	1.73 × 10^−6^	1.27 × 10^−4^	18
mitochondrial outer membrane	6.83 × 10^−4^	3.54 × 10^−2^	8
mitochondrial intermembrane space	8.05 × 10^−4^	3.54 × 10^−2^	6
lysosomal lumen	0.001504	4.80 × 10^−2^	6
cytosol	0.001528	4.80 × 10^−2^	50
endoplasmic reticulum-Golgi intermediate compartment	0.004495	0.124	5
mitochondrial matrix	0.005137	0.126	10
membrane	0.008466	0.186	34
peroxisomal matrix	0.010380	0.208	4
mitochondrial respiratory chain complex I	0.012331	0.226	4
**RNA_seq Down 24 h oxLDL**			
Term Name	Raw *P*Value	FDR *Q*-Value	Count
MHC class II protein complex	1.19 × 10^−5^	0.0021	5
integral component of lumenal side of endoplasmic reticulum membrane	3.74 × 10^−5^	0.0032	5
transport vesicle membrane	1.11 × 10^−4^	0.0064	5
clathrin-coated endocytic vesicle membrane	1.50 × 10^−4^	0.0064	5
ER to Golgi transport vesicle membrane	3.79 × 10^−4^	0.0130	5
cytoplasm	7.93 × 10^−4^	0.0227	52
endocytic vesicle membrane	9.41 × 10^−4^	0.0231	5
trans-Golgi network membrane	0.002201	0.0473	5
late endosome membrane	0.004467	0.0854	5
nuclear speck	0.010654	0.1833	6
Golgi membrane	0.016207	0.2534	10
lysosomal lumen	0.018599	0.2666	4

**Table 2:** Untargeted metabolomics pathway analysis shows intracellular enrichment of TCA cycle metabolites in oxLDL-trained macrophages. Table showing the results of metabolite set enrichment analysis (MSEA) of unannotated metabolites (*p* values for hypergeometric tests FET and EASE score, Gamma denotes the significance of permutation tests; *n* = 3).

Pathway	FET	EASE	Gamma
TCA cycle	0.019387	0.076825	0.033186
Alanine and Aspartate Metabolism	0.046403	0.12626	0.035023
C5-Branched dibasic acid metabolism	0.018149	0.12933	0.035142
Pyruvate Metabolism	0.060436	0.17042	0.036791
Methionine and cysteine metabolism	0.11386	0.2431	0.039995
Butanoate metabolism	0.10989	0.25753	0.040681
Glyoxylate and Dicarboxylate Metabolism	0.075442	0.28036	0.041802
Fatty acid oxidation, peroxisome	0.047007	0.34259	0.045107
Urea cycle/amino group metabolism	0.2352	0.40674	0.04896
Glycosphingolipid biosynthesis–ganglio series	0.16612	0.43069	0.050534
