# Supplementary material for: oxLDL-Induced Trained Immunity Is Dependent on Mitochondrial Metabolic Reprogramming
Source: Immunometabolism. Author manuscript; Available in PMC 2021 Jul 14. (PMC7611242; doi:10.20900/immunometab20210025)
Supplement: supplementary materials [file EMS129481-supplement-supplementary_materials.pdf]

Supplementary Materials

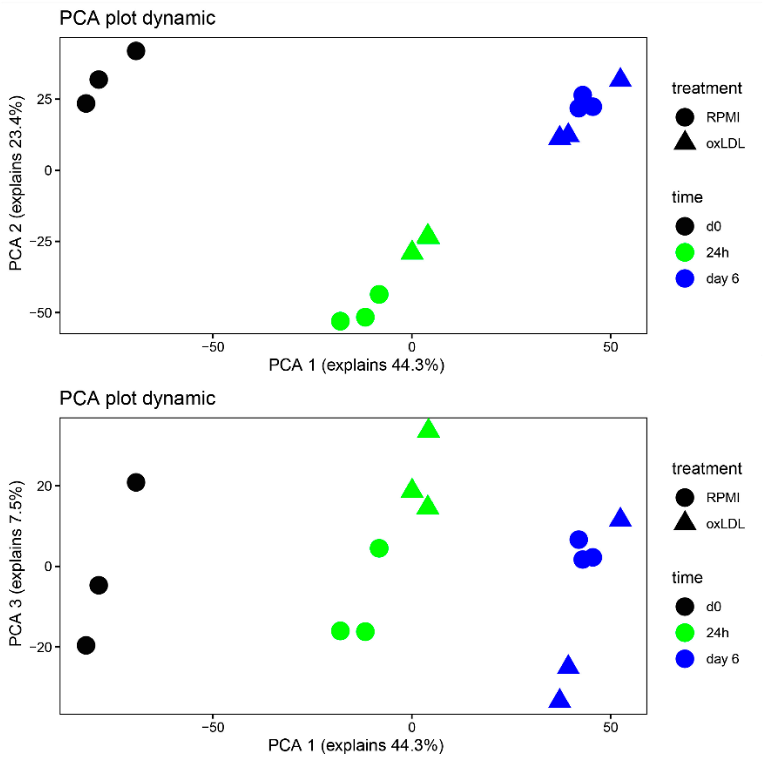

Supplementary Figure S1. Principal component analysis (PCA) plot of RNA-seq data (n = 3).

ID	Metabolite
C00122	Fumarate
C00149	L-Malate
C00026	2-Oxoglutarate
C00390	Ubiquinol
C00579	Dihydrolipoamide
C00311	Isocitrate
C00036	Oxaloacetate
C00158	Citrate
C00033	Acetate
C00074	Phosphoenolpyruvate
C00035	GDP
C00042	Succinate
C00044	GTP

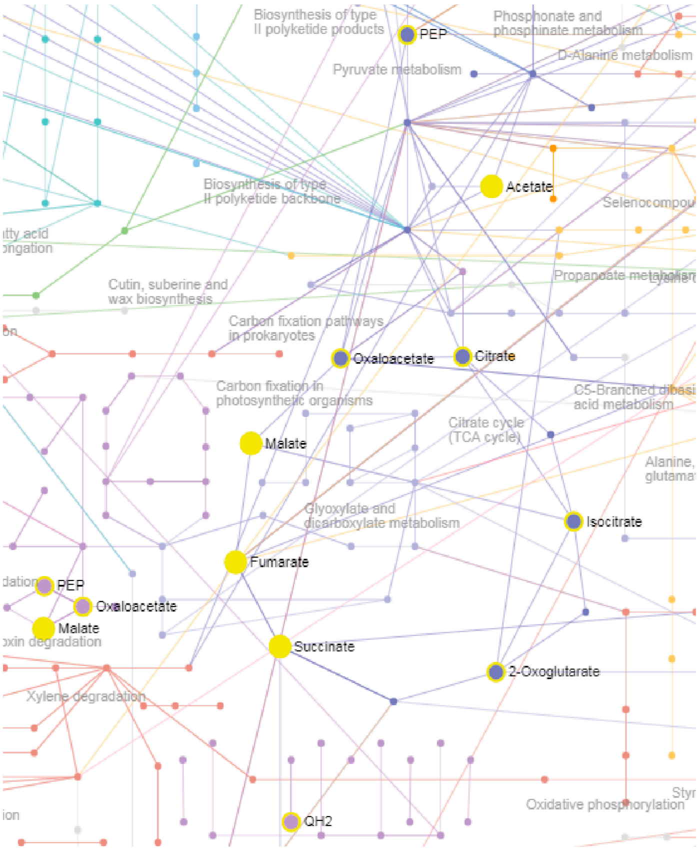

Supplementary Figure S2. List of identified tricarboxylic acid cycle (TCA) metabolites from Metabolomics pathway analysis.

Supplementary Table S1. Gene ID enriched in 'mitochondrion' pathway 24 h oxLDL.

ID	Gene Name
ENSG00000074696	3-hydroxyacyl-CoA dehydratase 3(HACD3)
ENSG00000138363	5-aminoimidazole-4-carboxamide ribonucleotide formyltransferase/IMP cyclohydrolase(ATIC)
ENSG00000241837	ATP synthase, H+ transporting, mitochondrial F1 complex, O subunit(ATP5O)
ENSG00000138495	COX17, cytochrome c oxidase copper chaperone(COX17)
ENSG00000007923	DnaJ heat shock protein family (Hsp40) member C11(DNAJC11)
ENSG00000132463	G-rich RNA sequence binding factor 1(GRSF1)
ENSG00000119421	NADH:ubiquinone oxidoreductase subunit A8(NDUFA8)
ENSG00000183648	NADH:ubiquinone oxidoreductase subunit B1(NDUFB1)
ENSG00000165264	NADH:ubiquinone oxidoreductase subunit B6(NDUFB6)
ENSG00000099795	NADH:ubiquinone oxidoreductase subunit B7(NDUFB7)
ENSG00000119705	SRA stem-loop interacting RNA binding protein(SLIRP)
ENSG00000119673	acyl-CoA thioesterase 2(ACOT2)
ENSG00000121691	catalase(CAT)
ENSG00000250479	coiled-coil-helix-coiled-coil-helix domain containing 10(CHCHD10)
ENSG00000164919	cytochrome c oxidase subunit 6C(COX6C)
ENSG00000131174	cytochrome c oxidase subunit 7B(COX7B)
ENSG00000088986	dynein light chain LC8-type 1(DYNLL1)
ENSG00000140374	electron transfer flavoprotein alpha subunit(ETFA)
ENSG00000114023	family with sequence similarity 162 member A(FAM162A)
ENSG00000091483	fumarate hydratase(FH)
ENSG00000096384	heat shock protein 90 alpha family class B member 1(HSP90AB1)
ENSG00000144381	heat shock protein family D (Hsp60) member 1(HSPD1)
ENSG00000138029	hydroxyacyl-CoA dehydrogenase/3-ketoacyl-CoA thiolase/enoyl-CoA hydratase (trifunctional protein), beta subunit(HADHB)
ENSG00000138413	isocitrate dehydrogenase (NADP(+)) 1, cytosolic(IDH1)
ENSG00000146701	malate dehydrogenase 2(MDH2)
ENSG00000100714	methylenetetrahydrofolate dehydrogenase, cyclohydrolase and formyltetrahydrofolate synthetase 1(MTHFD1)
ENSG00000008394	microsomal glutathione S-transferase 1(MGST1)
ENSG00000109919	mitochondrial carrier 2(MTCH2)
ENSG00000165672	peroxiredoxin 3(PRDX3)
ENSG00000100889	phosphoenolpyruvate carboxykinase 2, mitochondrial(PCK2)
ENSG00000114054	propionyl-CoA carboxylase beta subunit(PCCB)
ENSG00000137824	regulator of microtubule dynamics 3(RMDN3)
ENSG00000135002	riboflavin kinase(RFK)
ENSG00000143653	saccharopine dehydrogenase (putative)(SCCPDH)
ENSG00000168273	small integral membrane protein 4(SMIM4)
ENSG00000116171	sterol carrier protein 2(SCP2)
ENSG00000154174	translocase of outer mitochondrial membrane 70(TOMM70)

Supplementary Table S2. KEGG pathway analysis shows an increase in genes related to metabolism 24 hours after oxLDL treatment. Tables showing the pathways of the most up and down regulated genes following 24 h of oxLDL (10 μg/mL) treatment (differential gene expression calculated against unstimulated controls; *n* = 3).

**Upregulated**				
Term name	Count	%	*P*-Value	Benjamini
Biosynthesis of antibiotics	17	9.0	7.89E-07	1.21E-11
Metabolic pathways	40	21.3	8.66E-08	6.67E-10
Pyruvate metabolism	7	3.7	2.47E-11	0.001
PPAR signaling pathway	8	4.3	5.72E-11	0.002
Glutathione metabolism	7	3.7	1.02E-12	0.003
Oxidative phosphorylation	9	4.8	8.05E-11	0.021
Carbon metabolism	8	4.3	0.001	0.032
Alzheimer's disease	9	4.8	0.004	0.068
Parkinson's disease	8	4.3	0.005	0.090
Pentose phosphate pathway	4	2.1	0.009	0.138
Citrate cycle (TCA cycle)	4	2.1	0.010	0.138
**Downregulated**				
Term name	Count	%	*P*-value	Benjamini
Staphylococcus aureus infection	6	4.7	3.62E-10	0.004
Antigen processing and presentation	6	4.7	1.88E-11	0.010
Intestinal immune network for IgA production	5	3.9	3.24E-11	0.010
Rheumatoid arthritis	6	4.7	3.74E-11	0.010
Inflammatory bowel disease (IBD)	5	3.9	0.001	0.017
Asthma	4	3.1	0.001	0.017
Influenza A	7	5.4	0.001	0.017
Tuberculosis	7	5.4	0.001	0.017
Leishmaniasis	5	3.9	0.002	0.017
Graft-versus-host disease	4	3.1	0.002	0.017
Allograft rejection	4	3.1	0.002	0.021
